# 1,1′-[*o*-Phenyl­enebis(nitrilo­methyl­idyne)]di-2-naphthol ethanol hemisolvate

**DOI:** 10.1107/S1600536808021740

**Published:** 2008-07-19

**Authors:** Tuan-Jie Meng, Xiao-Qiang Qin, Wen-Xian Zhao, Xian-Qiang Huang, Guo-Dong Wei

**Affiliations:** a Department of Chemistry, Shangqiu Normal University, Shangqiu, Henan Province 476000, People’s Republic of China; b Department of Chemistry, Weifang Medical University, Weifang, Shandong Province 261053, People’s Republic of China; c Department of Chemistry, Liaocheng University, Liaocheng 252059, People’s Republic of China; d Shandong Donge Experimental High School, Donge, Shandong Province 252200, People’s Republic of China

## Abstract

The asymmetric unit of the title compound, C_28_H_20_N_2_O_2_·0.5C_2_H_5_OH, contains two independent mol­ecules of 1,1′-[*o*-phenyl­enebis(nitrilo­methyl­idyne)]di-2-naphthol, denoted A and B, and one ethanol solvent mol­ecule. The hydr­oxy groups are involved in intra­molecular O—H⋯N hydrogen bonds influencing the mol­ecular conformations, which are slightly different in mol­ecules A and B, where the two bicyclic systems form dihedral angles of 51.93 (9) and 58.52 (9)°, respectively. In the crystal structure, a number of short inter­molecular C⋯C contacts with distances of less than 3.5 Å suggest the existence of π–π inter­actions, which contribute to the stability of the crystal packing.

## Related literature

For related crystal structures, see: Zhang *et al.* (1990 ▶); Lo *et al.* (2006 ▶); Eltayeb *et al.* (2007 ▶).
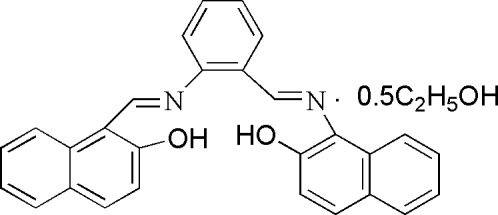


## Experimental

### 

#### Crystal data

C_28_H_20_N_2_O_2_·0.5C_2_H_6_O
*M* *_r_* = 439.50
Orthorhombic, 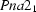

*a* = 19.956 (2) Å
*b* = 12.4742 (13) Å
*c* = 18.189 (2) Å
*V* = 4527.9 (8) Å^3^
*Z* = 8
Mo *K*α radiation
μ = 0.08 mm^−1^
*T* = 298 (2) K
0.20 × 0.18 × 0.07 mm

#### Data collection

Bruker SMART CCD area-detector diffractometer
Absorption correction: multi-scan (*SADABS*; Sheldrick, 1996 ▶) *T*_min_ = 0.984, *T*_max_ = 0.994
21755 measured reflections
4002 independent reflections
1545 reflections with *I* > 2σ(*I*)
*R*_int_ = 0.176

#### Refinement

*R*[*F* ^2^ > 2σ(*F* ^2^)] = 0.094
*wR*(*F* ^2^) = 0.121
*S* = 1.05
4002 reflections
605 parameters
1 restraint
H-atom parameters constrained
Δρ_max_ = 0.15 e Å^−3^
Δρ_min_ = −0.16 e Å^−3^

### 

Data collection: *SMART* (Siemens, 1996 ▶); cell refinement: *SAINT* (Siemens, 1996 ▶); data reduction: *SAINT*; program(s) used to solve structure: *SHELXS97* (Sheldrick, 2008 ▶); program(s) used to refine structure: *SHELXL97* (Sheldrick, 2008 ▶); molecular graphics: *SHELXTL* (Sheldrick, 2008 ▶); software used to prepare material for publication: *SHELXTL*.

## Supplementary Material

Crystal structure: contains datablocks I, global. DOI: 10.1107/S1600536808021740/cv2421sup1.cif

Structure factors: contains datablocks I. DOI: 10.1107/S1600536808021740/cv2421Isup2.hkl

Additional supplementary materials:  crystallographic information; 3D view; checkCIF report

## Figures and Tables

**Table 1:** Selected interatomic distances (Å)

C8⋯C35	3.485 (16)
C12⋯C34	3.387 (16)
C15⋯C46	3.473 (15)
C1⋯C54^i^	3.462 (17)
C7⋯C52^i^	3.481 (15)
C9⋯C47^i^	3.402 (16)
C13⋯C49^i^	3.475 (16)
C19⋯C37^ii^	3.418 (15)
C26⋯C29^ii^	3.308 (17)

Symmetry codes: (i) 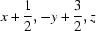; (ii) 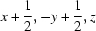.

**Table 2:** Hydrogen-bond geometry (Å, °)

*D*—H⋯*A*	*D*—H	H⋯*A*	*D*⋯*A*	*D*—H⋯*A*
O5—H5⋯O3	0.82	1.99	2.790 (11)	166
O4—H4⋯N4	0.82	1.87	2.594 (11)	147
O3—H3⋯N3	0.82	1.81	2.550 (10)	149
O2—H2⋯N2	0.82	1.85	2.578 (10)	148
O1—H1⋯N1	0.82	1.79	2.535 (9)	149

**Fig. 1.**
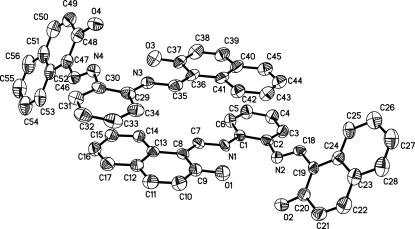
The content of asymmetric unit of the title compound showing the atomic numbering scheme and 30% probability displacement ellipsoids. Hydrogen atoms and solvent molecule omitted for clarity.

## supplementary crystallographic information

### Comment

Salen-type ligands are amongst the oldest ligands in coordination chemistry and have received considerable interest since Jacobsen and Katsuki first reported their significant success using chiral manganese (III) salen Schiff base catalysts in the asymmetric epoxidation of unfunctionalized olefins (Zhang *et al.*, 1990). In this paper, we report the crystal structure of the title compound, (I), obtained by the reaction of *o*-phenylenediamine and 2-hydroxy-1- naphthaldehyde.

All bond lengths and angles in (I) have normal values (Eltayeb *et al.*, 2007). The asymmetric unit of (I) contain two independent molecules (A and B) and one ethanol solvent molecule (Fig. 1). In A, the dihedral angles C12-C17/C1-C10, C12-C17/C19-C28 and C1-C10/C19-C28 are 4.71 (9), 51.28 (9) and 55.97 (7) °, respectively. In B, the dihedral angles C40-C45/C29-C38, C40-C45/C48-C56 and C29-C38/C48-C56 are 1.80 (9), 58.29 (9) and 59.84 (6) °, respectively. The hydroxyl groups are involved in intramolecular O—H···N hydrogen bonds (Table 2) influencing the molecular conformations.

In the crystal, a number of short intermolecular C···C contacts with the distances less than 3.5 Å (Table 1) suggest an existence of π-π interactions, which contribute to the crystal packing stability.

### Experimental

To a solution of *o*-phenylenediamine (3 mmol) in ethanol (30 ml) was added 2-hydroxy-1-naphthaldehyde (6 mmol). The mixture was refluxed with stirring for 20 min. An orange precipitate was then obtained. Red crystals suitable for X-ray diffraction analysis formed after several weeks on slow evaporation of a ethanol solution at room temperature. Elemental analysis: calculated for C_58_H_46_N_4_O_5_: C 79.25, H 5.27, N 6.37%; found: C 79.28, H 5.22, N 6.45%.

### Refinement

All H atoms were positioned geometrically and refined using a riding model with C—H = 0.93–0.98 Å and *U*_iso_(H) = 1.2-1.5 *U*_eq_(C). The H atoms of hydroxyl were placed in idealized positions , O—H 0.82%/A, the *U*_iso_(H) values were set at 1.5 *U*_eq_(*O*). In the absence of any significant anomalous scatterers in the compound, the 3833 Friedel pairs were merged before the final refinement.

### Crystal data

**Table:**

C_28_H_20_N_2_O_2_·0.5(C_2_H_6_O)	*D*_x_ = 1.289 Mg m^−^^3^
*M**_r_* = 439.50	Mo *K*α radiation λ = 0.71073 Å
Orthorhombic, *P**n**a*2_1_	Cell parameters from 1646 reflections
*a* = 19.956 (2) Å	θ = 2.9–28.1º
*b* = 12.4742 (13) Å	µ = 0.08 mm^−^^1^
*c* = 18.189 (2) Å	*T* = 298 (2) K
*V* = 4527.9 (8) Å^3^	Block, yellow
*Z* = 8	0.20 × 0.18 × 0.07 mm
*F*_000_ = 1848	

### Data collection

**Table:**

Bruker SMART CCD area-detector diffractometer	4002 independent reflections
Radiation source: fine-focus sealed tube	1545 reflections with *I* > 2σ(*I*)
Monochromator: graphite	*R*_int_ = 0.176
*T* = 298(2) K	θ_max_ = 25.0º
φ and ω scans	θ_min_ = 1.9º
Absorption correction: multi-scan(SADABS; Sheldrick, 1996)	*h* = −19→23
*T*_min_ = 0.984, *T*_max_ = 0.994	*k* = −14→14
21755 measured reflections	*l* = −21→21

### Refinement

**Table:**

Refinement on *F*^2^	Secondary atom site location: difference Fourier map
Least-squares matrix: full	Hydrogen site location: inferred from neighbouring sites
*R*[*F*^2^ > 2σ(*F*^2^)] = 0.094	H-atom parameters constrained
*wR*(*F*^2^) = 0.121	*w* = 1/[σ^2^(*F*_o_^2^) + (0.026*P*)^2^] where *P* = (*F*_o_^2^ + 2*F*_c_^2^)/3
*S* = 1.06	(Δ/σ)_max_ = 0.001
4002 reflections	Δρ_max_ = 0.15 e Å^−^^3^
605 parameters	Δρ_min_ = −0.16 e Å^−^^3^
1 restraint	Extinction correction: none
Primary atom site location: structure-invariant direct methods	

### Special details

**Table:**

Geometry. All e.s.d.'s (except the e.s.d. in the dihedral angle between two l.s. planes) are estimated using the full covariance matrix. The cell e.s.d.'s are taken into account individually in the estimation of e.s.d.'s in distances, angles and torsion angles; correlations between e.s.d.'s in cell parameters are only used when they are defined by crystal symmetry. An approximate (isotropic) treatment of cell e.s.d.'s is used for estimating e.s.d.'s involving l.s. planes.
Refinement. Refinement of *F*^2^ against ALL reflections. The weighted *R*-factor *wR* and goodness of fit *S* are based on *F*^2^, conventional *R*-factors *R* are based on *F*, with *F* set to zero for negative *F*^2^. The threshold expression of *F*^2^ > σ(*F*^2^) is used only for calculating *R*-factors(gt) *etc*. and is not relevant to the choice of reflections for refinement. *R*-factors based on *F*^2^ are statistically about twice as large as those based on *F*, and *R*- factors based on ALL data will be even larger.

### Fractional atomic coordinates and isotropic or equivalent isotropic displacement parameters (Å^2^)

**Table:**

	*x*	*y*	*z*	*U*_iso_*/*U*_eq_	
N1	0.4622 (4)	0.4463 (5)	0.2203 (4)	0.061 (2)	
N2	0.5628 (4)	0.3113 (6)	0.1967 (4)	0.065 (2)	
N3	0.2294 (4)	0.5681 (6)	0.1085 (4)	0.061 (2)	
N4	0.1288 (4)	0.7009 (6)	0.1347 (5)	0.069 (2)	
O1	0.5069 (4)	0.5421 (5)	0.1076 (4)	0.087 (2)	
H1	0.5021	0.4946	0.1383	0.130*	
O2	0.6557 (3)	0.4250 (5)	0.1367 (4)	0.088 (2)	
H2	0.6266	0.4091	0.1666	0.132*	
O3	0.1832 (4)	0.4657 (5)	0.2186 (4)	0.090 (2)	
H3	0.1863	0.5130	0.1876	0.136*	
O4	0.0335 (4)	0.5789 (5)	0.1839 (4)	0.090 (3)	
H4	0.0640	0.5976	0.1565	0.136*	
O5	0.1992 (5)	0.5525 (9)	0.3589 (5)	0.175 (5)	
H5	0.1973	0.5175	0.3208	0.263*	
C1	0.4667 (5)	0.3606 (7)	0.2677 (5)	0.058 (3)	
C2	0.5175 (6)	0.2856 (7)	0.2548 (5)	0.065 (3)	
C3	0.5266 (6)	0.1963 (8)	0.2979 (6)	0.081 (3)	
H3A	0.5622	0.1499	0.2890	0.097*	
C4	0.4820 (6)	0.1766 (8)	0.3547 (6)	0.083 (3)	
H4A	0.4862	0.1145	0.3826	0.100*	
C5	0.4317 (6)	0.2479 (8)	0.3701 (6)	0.085 (4)	
H5A	0.4033	0.2357	0.4098	0.102*	
C6	0.4232 (6)	0.3379 (8)	0.3269 (5)	0.075 (3)	
H6	0.3881	0.3845	0.3370	0.090*	
C7	0.4192 (5)	0.5246 (7)	0.2259 (5)	0.058 (3)	
H7	0.3892	0.5234	0.2650	0.070*	
C8	0.4156 (6)	0.6088 (8)	0.1773 (6)	0.061 (3)	
C9	0.4628 (6)	0.6155 (8)	0.1181 (7)	0.069 (3)	
C10	0.4633 (6)	0.7026 (9)	0.0693 (6)	0.081 (3)	
H10	0.4929	0.7036	0.0299	0.097*	
C11	0.4208 (7)	0.7840 (9)	0.0800 (7)	0.085 (4)	
H11	0.4235	0.8436	0.0493	0.102*	
C12	0.3710 (6)	0.7826 (8)	0.1374 (6)	0.070 (3)	
C13	0.3675 (5)	0.6957 (8)	0.1864 (6)	0.059 (3)	
C14	0.3169 (6)	0.6970 (9)	0.2400 (5)	0.067 (3)	
H14	0.3126	0.6395	0.2723	0.081*	
C15	0.2741 (6)	0.7811 (9)	0.2454 (6)	0.082 (4)	
H15	0.2405	0.7791	0.2809	0.099*	
C16	0.2785 (6)	0.8696 (9)	0.2000 (7)	0.087 (4)	
H16	0.2497	0.9277	0.2056	0.104*	
C17	0.3271 (6)	0.8687 (9)	0.1464 (6)	0.082 (4)	
H17	0.3308	0.9274	0.1151	0.098*	
C18	0.5801 (5)	0.2358 (8)	0.1518 (5)	0.057 (3)	
H18	0.5614	0.1680	0.1578	0.069*	
C19	0.6269 (5)	0.2526 (7)	0.0933 (5)	0.053 (3)	
C20	0.6634 (6)	0.3443 (8)	0.0881 (6)	0.070 (3)	
C21	0.7127 (6)	0.3604 (10)	0.0342 (7)	0.090 (4)	
H21	0.7369	0.4242	0.0329	0.108*	
C22	0.7249 (7)	0.2819 (12)	−0.0164 (7)	0.097 (4)	
H22	0.7582	0.2917	−0.0516	0.116*	
C23	0.6875 (6)	0.1859 (11)	−0.0156 (6)	0.079 (3)	
C24	0.6383 (6)	0.1729 (8)	0.0383 (6)	0.064 (3)	
C25	0.6016 (6)	0.0770 (9)	0.0364 (5)	0.077 (3)	
H25	0.5687	0.0650	0.0717	0.093*	
C26	0.6131 (7)	0.0004 (9)	−0.0165 (7)	0.091 (4)	
H26	0.5871	−0.0615	−0.0167	0.109*	
C27	0.6626 (8)	0.0126 (11)	−0.0696 (7)	0.112 (5)	
H27	0.6701	−0.0399	−0.1050	0.134*	
C28	0.6992 (8)	0.1034 (12)	−0.0680 (6)	0.104 (5)	
H28	0.7333	0.1124	−0.1022	0.125*	
C29	0.2245 (6)	0.6585 (8)	0.0626 (5)	0.061 (3)	
C30	0.1732 (6)	0.7312 (8)	0.0783 (6)	0.061 (3)	
C31	0.1655 (6)	0.8246 (8)	0.0391 (5)	0.073 (3)	
H31	0.1312	0.8723	0.0503	0.087*	
C32	0.2102 (7)	0.8460 (9)	−0.0176 (7)	0.092 (4)	
H32	0.2059	0.9091	−0.0445	0.111*	
C33	0.2607 (8)	0.7748 (11)	−0.0346 (6)	0.099 (5)	
H33	0.2894	0.7896	−0.0735	0.119*	
C34	0.2692 (6)	0.6825 (10)	0.0052 (6)	0.084 (4)	
H34	0.3043	0.6361	−0.0057	0.101*	
C35	0.2738 (6)	0.4901 (9)	0.1006 (5)	0.070 (3)	
H35	0.3048	0.4940	0.0625	0.085*	
C36	0.2755 (5)	0.4010 (10)	0.1486 (6)	0.065 (3)	
C37	0.2286 (6)	0.3932 (9)	0.2067 (6)	0.070 (3)	
C38	0.2295 (7)	0.3021 (11)	0.2539 (6)	0.095 (4)	
H38	0.1984	0.2963	0.2918	0.114*	
C39	0.2755 (7)	0.2246 (10)	0.2437 (7)	0.096 (4)	
H39	0.2760	0.1669	0.2760	0.115*	
C40	0.3226 (6)	0.2265 (11)	0.1868 (7)	0.082 (3)	
C41	0.3239 (6)	0.3154 (10)	0.1385 (6)	0.070 (3)	
C42	0.3731 (7)	0.3146 (9)	0.0840 (7)	0.090 (4)	
H42	0.3755	0.3720	0.0515	0.107*	
C43	0.4186 (6)	0.2309 (11)	0.0766 (9)	0.108 (5)	
H43	0.4513	0.2328	0.0403	0.130*	
C44	0.4144 (7)	0.1455 (12)	0.1239 (9)	0.110 (5)	
H44	0.4440	0.0884	0.1188	0.132*	
C45	0.3684 (7)	0.1434 (11)	0.1771 (8)	0.100 (5)	
H45	0.3668	0.0848	0.2087	0.120*	
C46	0.1101 (5)	0.7724 (7)	0.1818 (5)	0.062 (3)	
H46	0.1284	0.8409	0.1790	0.074*	
C47	0.0611 (6)	0.7490 (8)	0.2394 (5)	0.066 (3)	
C48	0.0233 (6)	0.6535 (10)	0.2340 (6)	0.072 (3)	
C49	−0.0279 (6)	0.6367 (9)	0.2871 (8)	0.089 (4)	
H49	−0.0544	0.5755	0.2842	0.107*	
C50	−0.0385 (7)	0.7058 (12)	0.3396 (6)	0.104 (5)	
H50	−0.0734	0.6908	0.3718	0.124*	
C51	−0.0030 (8)	0.7987 (10)	0.3522 (7)	0.095 (4)	
C52	0.0501 (6)	0.8240 (9)	0.2981 (6)	0.076 (3)	
C53	0.0881 (6)	0.9171 (10)	0.3089 (6)	0.084 (4)	
H53	0.1216	0.9349	0.2755	0.101*	
C54	0.0765 (8)	0.9826 (9)	0.3685 (8)	0.132 (6)	
H54	0.1031	1.0429	0.3757	0.159*	
C55	0.0237 (10)	0.9590 (13)	0.4198 (7)	0.134 (7)	
H55	0.0144	1.0041	0.4592	0.161*	
C56	−0.0111 (9)	0.8695 (13)	0.4085 (8)	0.124 (6)	
H56	−0.0445	0.8538	0.4425	0.148*	
C57	0.1328 (10)	0.5731 (16)	0.3850 (11)	0.188 (8)	
H57A	0.1299	0.6484	0.3977	0.226*	
H57B	0.1020	0.5608	0.3446	0.226*	
C58	0.1125 (10)	0.5189 (14)	0.4398 (11)	0.220 (9)	
H58A	0.1198	0.4440	0.4307	0.329*	
H58B	0.0656	0.5317	0.4473	0.329*	
H58C	0.1369	0.5402	0.4830	0.329*	

### Atomic displacement parameters (Å^2^)

**Table:**

	*U*^11^	*U*^22^	*U*^33^	*U*^12^	*U*^13^	*U*^23^
N1	0.086 (7)	0.021 (4)	0.074 (5)	0.002 (4)	−0.003 (5)	0.004 (4)
N2	0.056 (6)	0.066 (5)	0.073 (6)	0.002 (5)	0.004 (5)	−0.008 (5)
N3	0.061 (6)	0.059 (5)	0.062 (5)	−0.004 (5)	−0.003 (5)	−0.008 (5)
N4	0.069 (7)	0.068 (5)	0.070 (6)	−0.007 (5)	0.004 (6)	0.000 (5)
O1	0.095 (6)	0.086 (5)	0.079 (5)	0.006 (5)	0.019 (5)	0.007 (4)
O2	0.068 (6)	0.073 (4)	0.123 (6)	−0.013 (4)	0.019 (5)	0.005 (5)
O3	0.087 (6)	0.113 (5)	0.070 (5)	0.013 (5)	0.007 (5)	0.010 (4)
O4	0.095 (7)	0.083 (5)	0.093 (5)	−0.020 (5)	0.004 (5)	−0.003 (5)
O5	0.127 (9)	0.277 (13)	0.122 (8)	−0.038 (10)	0.012 (8)	−0.059 (8)
C1	0.059 (8)	0.043 (6)	0.073 (7)	−0.003 (6)	0.007 (6)	−0.020 (6)
C2	0.079 (9)	0.050 (6)	0.065 (7)	−0.017 (6)	0.007 (7)	0.007 (6)
C3	0.072 (9)	0.075 (8)	0.096 (9)	0.012 (7)	0.012 (8)	0.010 (7)
C4	0.081 (9)	0.088 (8)	0.081 (8)	0.011 (7)	0.016 (7)	0.030 (7)
C5	0.093 (10)	0.093 (9)	0.070 (8)	0.011 (7)	0.036 (7)	0.013 (7)
C6	0.085 (9)	0.078 (8)	0.063 (7)	0.011 (7)	0.013 (7)	−0.002 (6)
C7	0.076 (8)	0.041 (5)	0.058 (6)	−0.012 (6)	0.000 (6)	−0.004 (5)
C8	0.066 (9)	0.057 (7)	0.060 (7)	−0.013 (6)	0.002 (7)	−0.009 (6)
C9	0.074 (10)	0.057 (7)	0.077 (8)	0.003 (7)	−0.016 (8)	−0.014 (7)
C10	0.083 (11)	0.081 (8)	0.077 (8)	0.000 (8)	0.004 (7)	0.002 (7)
C11	0.094 (11)	0.086 (9)	0.075 (9)	−0.015 (8)	−0.007 (8)	0.013 (7)
C12	0.064 (9)	0.073 (7)	0.072 (8)	−0.001 (7)	−0.022 (7)	−0.011 (7)
C13	0.065 (8)	0.057 (6)	0.054 (6)	−0.007 (6)	−0.022 (7)	−0.004 (6)
C14	0.061 (8)	0.076 (7)	0.066 (7)	0.001 (7)	−0.006 (7)	−0.015 (6)
C15	0.068 (10)	0.084 (8)	0.094 (9)	−0.001 (8)	−0.013 (7)	−0.025 (8)
C16	0.080 (10)	0.070 (8)	0.110 (10)	0.011 (7)	−0.021 (9)	−0.020 (8)
C17	0.079 (10)	0.083 (8)	0.082 (9)	−0.001 (8)	−0.014 (8)	0.007 (7)
C18	0.047 (7)	0.062 (6)	0.062 (7)	−0.007 (6)	−0.002 (6)	0.021 (6)
C19	0.062 (8)	0.032 (5)	0.066 (7)	0.014 (5)	0.005 (6)	0.007 (5)
C20	0.074 (9)	0.044 (6)	0.091 (8)	0.009 (6)	0.008 (7)	0.006 (6)
C21	0.075 (10)	0.088 (9)	0.107 (10)	−0.007 (8)	0.024 (8)	0.031 (8)
C22	0.090 (11)	0.109 (11)	0.091 (10)	0.010 (9)	0.019 (9)	0.037 (8)
C23	0.075 (9)	0.109 (10)	0.053 (7)	0.023 (8)	0.016 (7)	0.028 (7)
C24	0.064 (8)	0.050 (6)	0.077 (8)	0.008 (6)	−0.009 (7)	0.021 (6)
C25	0.098 (10)	0.083 (8)	0.050 (6)	0.041 (7)	−0.010 (6)	−0.004 (6)
C26	0.104 (11)	0.094 (9)	0.074 (8)	0.008 (8)	−0.025 (8)	0.000 (8)
C27	0.153 (17)	0.113 (11)	0.069 (9)	0.035 (11)	−0.011 (10)	−0.013 (9)
C28	0.129 (14)	0.130 (11)	0.052 (7)	0.044 (11)	0.015 (8)	0.001 (9)
C29	0.076 (9)	0.058 (6)	0.050 (6)	−0.009 (6)	−0.011 (6)	−0.013 (6)
C30	0.070 (9)	0.058 (7)	0.053 (7)	−0.009 (6)	−0.004 (6)	−0.009 (6)
C31	0.091 (10)	0.074 (7)	0.053 (6)	−0.015 (6)	0.002 (7)	0.009 (6)
C32	0.125 (12)	0.080 (9)	0.072 (8)	−0.024 (9)	−0.020 (9)	−0.005 (7)
C33	0.135 (15)	0.099 (10)	0.064 (8)	−0.042 (10)	0.003 (8)	0.001 (8)
C34	0.083 (10)	0.105 (10)	0.065 (7)	−0.024 (8)	0.021 (7)	−0.012 (7)
C35	0.070 (9)	0.085 (7)	0.057 (7)	−0.018 (7)	0.004 (6)	−0.021 (6)
C36	0.030 (7)	0.098 (9)	0.066 (8)	−0.002 (7)	0.003 (6)	−0.013 (7)
C37	0.046 (8)	0.098 (9)	0.067 (8)	0.006 (7)	−0.015 (7)	−0.005 (7)
C38	0.096 (12)	0.126 (10)	0.062 (8)	0.000 (9)	−0.013 (8)	0.029 (8)
C39	0.087 (11)	0.108 (10)	0.091 (10)	0.000 (9)	−0.020 (9)	0.019 (8)
C40	0.067 (10)	0.093 (9)	0.085 (9)	0.000 (8)	−0.007 (8)	−0.011 (8)
C41	0.045 (8)	0.095 (9)	0.069 (8)	−0.009 (7)	0.002 (7)	−0.012 (7)
C42	0.085 (10)	0.079 (8)	0.105 (10)	−0.007 (8)	−0.005 (9)	−0.013 (7)
C43	0.068 (11)	0.093 (10)	0.163 (13)	0.009 (9)	0.000 (9)	−0.017 (10)
C44	0.072 (12)	0.096 (11)	0.161 (15)	0.010 (9)	−0.014 (11)	−0.032 (11)
C45	0.081 (12)	0.089 (9)	0.130 (14)	−0.009 (9)	−0.014 (10)	−0.006 (9)
C46	0.061 (8)	0.062 (6)	0.063 (7)	−0.005 (6)	−0.010 (6)	0.012 (6)
C47	0.061 (8)	0.076 (7)	0.060 (7)	0.005 (7)	−0.009 (6)	0.012 (6)
C48	0.060 (9)	0.093 (9)	0.063 (7)	0.002 (8)	−0.002 (7)	0.031 (7)
C49	0.064 (9)	0.098 (9)	0.105 (10)	0.006 (7)	−0.008 (9)	0.028 (8)
C50	0.118 (13)	0.123 (12)	0.070 (9)	0.050 (11)	0.026 (9)	0.015 (9)
C51	0.138 (14)	0.078 (10)	0.070 (9)	0.051 (9)	−0.003 (10)	0.004 (8)
C52	0.087 (10)	0.084 (9)	0.056 (7)	0.026 (8)	0.000 (7)	0.013 (7)
C53	0.098 (11)	0.085 (8)	0.069 (8)	0.028 (8)	−0.012 (7)	−0.007 (7)
C54	0.198 (18)	0.112 (11)	0.087 (9)	0.065 (11)	−0.040 (11)	−0.025 (9)
C55	0.21 (2)	0.130 (13)	0.060 (9)	0.080 (14)	0.012 (11)	−0.013 (10)
C56	0.176 (18)	0.116 (11)	0.078 (10)	0.077 (12)	0.017 (10)	0.023 (10)
C57	0.17 (2)	0.26 (2)	0.134 (17)	0.003 (19)	−0.054 (16)	0.041 (15)
C58	0.24 (2)	0.26 (2)	0.157 (17)	−0.066 (17)	−0.024 (17)	0.073 (14)

### Geometric parameters (Å, °)

**Table:**

N1—C7	1.304 (9)	C25—H25	0.9300
N1—C1	1.376 (10)	C26—C27	1.389 (16)
N2—C18	1.294 (10)	C26—H26	0.9300
N2—C2	1.426 (10)	C27—C28	1.349 (15)
N3—C35	1.324 (11)	C27—H27	0.9300
N3—C29	1.406 (10)	C28—H28	0.9300
N4—C46	1.292 (10)	C29—C30	1.398 (12)
N4—C30	1.408 (11)	C29—C34	1.405 (13)
O1—C9	1.284 (11)	C30—C31	1.374 (12)
O1—H1	0.8200	C31—C32	1.389 (14)
O2—C20	1.348 (10)	C31—H31	0.9300
O2—H2	0.8200	C32—C33	1.378 (14)
O3—C37	1.299 (10)	C32—H32	0.9300
O3—H3	0.8200	C33—C34	1.370 (14)
O4—C48	1.319 (12)	C33—H33	0.9300
O4—H4	0.8200	C34—H34	0.9300
O5—C57	1.43 (2)	C35—C36	1.414 (13)
O5—H5	0.8200	C35—H35	0.9300
C1—C2	1.400 (12)	C36—C37	1.415 (13)
C1—C6	1.411 (12)	C36—C41	1.452 (14)
C2—C3	1.374 (12)	C37—C38	1.424 (12)
C3—C4	1.387 (13)	C38—C39	1.347 (14)
C3—H3A	0.9300	C38—H38	0.9300
C4—C5	1.369 (12)	C39—C40	1.397 (14)
C4—H4A	0.9300	C39—H39	0.9300
C5—C6	1.381 (12)	C40—C45	1.392 (15)
C5—H5A	0.9300	C40—C41	1.415 (14)
C6—H6	0.9300	C41—C42	1.394 (13)
C7—C8	1.374 (12)	C42—C43	1.390 (14)
C7—H7	0.9300	C42—H42	0.9300
C8—C9	1.434 (14)	C43—C44	1.371 (16)
C8—C13	1.456 (13)	C43—H43	0.9300
C9—C10	1.402 (13)	C44—C45	1.335 (16)
C10—C11	1.337 (14)	C44—H44	0.9300
C10—H10	0.9300	C45—H45	0.9300
C11—C12	1.442 (14)	C46—C47	1.463 (12)
C11—H11	0.9300	C46—H46	0.9300
C12—C17	1.396 (13)	C47—C48	1.413 (13)
C12—C13	1.405 (12)	C47—C52	1.436 (13)
C13—C14	1.405 (12)	C48—C49	1.421 (14)
C14—C15	1.357 (12)	C49—C50	1.304 (13)
C14—H14	0.9300	C49—H49	0.9300
C15—C16	1.381 (12)	C50—C51	1.377 (16)
C15—H15	0.9300	C50—H50	0.9300
C16—C17	1.377 (14)	C51—C56	1.361 (14)
C16—H16	0.9300	C51—C52	1.480 (16)
C17—H17	0.9300	C52—C53	1.401 (14)
C18—C19	1.432 (12)	C53—C54	1.377 (15)
C18—H18	0.9300	C53—H53	0.9300
C19—C20	1.359 (12)	C54—C55	1.437 (18)
C19—C24	1.428 (12)	C54—H54	0.9300
C20—C21	1.404 (13)	C55—C56	1.332 (19)
C21—C22	1.366 (14)	C55—H55	0.9300
C21—H21	0.9300	C56—H56	0.9300
C22—C23	1.411 (14)	C57—C58	1.271 (18)
C22—H22	0.9300	C57—H57A	0.9700
C23—C24	1.398 (13)	C57—H57B	0.9700
C23—C28	1.421 (15)	C58—H58A	0.9600
C24—C25	1.403 (13)	C58—H58B	0.9600
C25—C26	1.377 (13)	C58—H58C	0.9600
			
C8···C35	3.485 (16)	C9···C47^i^	3.402 (16)
C12···C34	3.387 (16)	C13···C49^i^	3.475 (16)
C15···C46	3.473 (15)	C19···C37^ii^	3.418 (15)
C1···C54^i^	3.462 (17)	C26···C29^ii^	3.308 (17)
C7···C52^i^	3.481 (15)		
			
C7—N1—C1	125.2 (9)	C34—C29—N3	124.6 (11)
C18—N2—C2	118.2 (8)	C31—C30—C29	121.7 (11)
C35—N3—C29	124.9 (10)	C31—C30—N4	122.4 (11)
C46—N4—C30	118.6 (9)	C29—C30—N4	115.8 (9)
C9—O1—H1	109.5	C30—C31—C32	118.5 (11)
C20—O2—H2	109.5	C30—C31—H31	120.8
C37—O3—H3	109.5	C32—C31—H31	120.8
C48—O4—H4	109.5	C33—C32—C31	120.7 (12)
C57—O5—H5	109.5	C33—C32—H32	119.6
N1—C1—C2	117.5 (10)	C31—C32—H32	119.6
N1—C1—C6	126.4 (10)	C34—C33—C32	121.0 (13)
C2—C1—C6	116.1 (9)	C34—C33—H33	119.5
C3—C2—C1	122.8 (11)	C32—C33—H33	119.5
C3—C2—N2	121.4 (11)	C33—C34—C29	119.5 (12)
C1—C2—N2	115.6 (9)	C33—C34—H34	120.3
C2—C3—C4	118.9 (11)	C29—C34—H34	120.3
C2—C3—H3A	120.5	N3—C35—C36	121.8 (10)
C4—C3—H3A	120.5	N3—C35—H35	119.1
C5—C4—C3	120.6 (10)	C36—C35—H35	119.1
C5—C4—H4A	119.7	C35—C36—C37	120.0 (11)
C3—C4—H4A	119.7	C35—C36—C41	121.0 (11)
C4—C5—C6	120.1 (10)	C37—C36—C41	118.9 (11)
C4—C5—H5A	120.0	O3—C37—C36	122.6 (11)
C6—C5—H5A	120.0	O3—C37—C38	117.6 (12)
C5—C6—C1	121.5 (10)	C36—C37—C38	119.8 (12)
C5—C6—H6	119.3	C39—C38—C37	119.9 (13)
C1—C6—H6	119.3	C39—C38—H38	120.0
N1—C7—C8	123.9 (10)	C37—C38—H38	120.0
N1—C7—H7	118.0	C38—C39—C40	123.3 (13)
C8—C7—H7	118.0	C38—C39—H39	118.4
C7—C8—C9	119.5 (11)	C40—C39—H39	118.4
C7—C8—C13	122.1 (11)	C45—C40—C39	121.4 (15)
C9—C8—C13	118.3 (10)	C45—C40—C41	119.5 (13)
O1—C9—C10	116.9 (12)	C39—C40—C41	119.0 (13)
O1—C9—C8	121.4 (11)	C42—C41—C40	116.6 (12)
C10—C9—C8	121.7 (11)	C42—C41—C36	124.3 (12)
C11—C10—C9	119.5 (12)	C40—C41—C36	119.1 (12)
C11—C10—H10	120.3	C43—C42—C41	122.3 (13)
C9—C10—H10	120.3	C43—C42—H42	118.9
C10—C11—C12	122.2 (11)	C41—C42—H42	118.9
C10—C11—H11	118.9	C44—C43—C42	118.9 (14)
C12—C11—H11	118.9	C44—C43—H43	120.6
C17—C12—C13	119.3 (12)	C42—C43—H43	120.6
C17—C12—C11	120.5 (12)	C45—C44—C43	120.8 (16)
C13—C12—C11	120.2 (11)	C45—C44—H44	119.6
C12—C13—C14	117.8 (11)	C43—C44—H44	119.6
C12—C13—C8	118.1 (11)	C44—C45—C40	121.9 (15)
C14—C13—C8	124.1 (10)	C44—C45—H45	119.0
C15—C14—C13	120.8 (11)	C40—C45—H45	119.0
C15—C14—H14	119.6	N4—C46—C47	122.0 (9)
C13—C14—H14	119.6	N4—C46—H46	119.0
C14—C15—C16	122.3 (12)	C47—C46—H46	119.0
C14—C15—H15	118.8	C48—C47—C52	121.3 (11)
C16—C15—H15	118.8	C48—C47—C46	118.4 (10)
C17—C16—C15	117.5 (12)	C52—C47—C46	120.3 (11)
C17—C16—H16	121.2	O4—C48—C47	124.1 (11)
C15—C16—H16	121.2	O4—C48—C49	118.5 (12)
C16—C17—C12	122.1 (12)	C47—C48—C49	117.4 (12)
C16—C17—H17	118.9	C50—C49—C48	121.1 (13)
C12—C17—H17	118.9	C50—C49—H49	119.4
N2—C18—C19	122.5 (9)	C48—C49—H49	119.4
N2—C18—H18	118.8	C49—C50—C51	126.5 (14)
C19—C18—H18	118.8	C49—C50—H50	116.7
C20—C19—C24	116.9 (10)	C51—C50—H50	116.7
C20—C19—C18	121.6 (10)	C56—C51—C50	127.6 (17)
C24—C19—C18	121.5 (9)	C56—C51—C52	116.5 (14)
O2—C20—C19	121.5 (10)	C50—C51—C52	115.9 (12)
O2—C20—C21	115.5 (11)	C53—C52—C47	124.2 (12)
C19—C20—C21	123.0 (11)	C53—C52—C51	118.1 (12)
C22—C21—C20	119.5 (12)	C47—C52—C51	117.7 (12)
C22—C21—H21	120.2	C54—C53—C52	120.7 (13)
C20—C21—H21	120.2	C54—C53—H53	119.6
C21—C22—C23	120.5 (13)	C52—C53—H53	119.6
C21—C22—H22	119.8	C53—C54—C55	120.9 (14)
C23—C22—H22	119.8	C53—C54—H54	119.6
C24—C23—C22	118.5 (12)	C55—C54—H54	119.6
C24—C23—C28	120.1 (13)	C56—C55—C54	117.0 (14)
C22—C23—C28	121.4 (13)	C56—C55—H55	121.5
C23—C24—C25	116.6 (11)	C54—C55—H55	121.5
C23—C24—C19	121.5 (11)	C55—C56—C51	126.8 (17)
C25—C24—C19	121.9 (10)	C55—C56—H56	116.6
C26—C25—C24	121.5 (12)	C51—C56—H56	116.6
C26—C25—H25	119.3	C58—C57—O5	117.4 (19)
C24—C25—H25	119.3	C58—C57—H57A	108.0
C25—C26—C27	122.0 (13)	O5—C57—H57A	108.0
C25—C26—H26	119.0	C58—C57—H57B	108.0
C27—C26—H26	119.0	O5—C57—H57B	108.0
C28—C27—C26	117.5 (14)	H57A—C57—H57B	107.2
C28—C27—H27	121.3	C57—C58—H58A	109.5
C26—C27—H27	121.3	C57—C58—H58B	109.5
C27—C28—C23	122.3 (14)	H58A—C58—H58B	109.5
C27—C28—H28	118.9	C57—C58—H58C	109.5
C23—C28—H28	118.9	H58A—C58—H58C	109.5
C30—C29—C34	118.6 (10)	H58B—C58—H58C	109.5
C30—C29—N3	116.8 (10)		

Symmetry codes: (i) *x*+1/2, −*y*+3/2, *z*; (ii) *x*+1/2, −*y*+1/2, *z*.

### Hydrogen-bond geometry (Å, °)

**Table:**

*D*—H···*A*	*D*—H	H···*A*	*D*···*A*	*D*—H···*A*
O5—H5···O3	0.82	1.99	2.790 (11)	166
O4—H4···N4	0.82	1.87	2.594 (11)	147
O3—H3···N3	0.82	1.81	2.550 (10)	149
O2—H2···N2	0.82	1.85	2.578 (10)	148
O1—H1···N1	0.82	1.79	2.535 (9)	149
